# The genetic background of the associations between sense of coherence and mental health, self-esteem and personality

**DOI:** 10.1007/s00127-021-02098-6

**Published:** 2021-05-19

**Authors:** Karri Silventoinen, Eero Vuoksimaa, Salla-Maarit Volanen, Teemu Palviainen, Richard J. Rose, Sakari Suominen, Jaakko Kaprio

**Affiliations:** 1 Faculty of Social Sciences, Department of Social Research, University of Helsinki, P.O. Box 18, N00014 Helsinki, Finland; 2 Institute for Molecular Medicine Finland (FIMM), HiLIFE, University of Helsinki, Helsinki, Finland; 3 Folkhälsan Research Center, Helsinki, Finland; 4 Clinicum, Department of Public Health, University of Helsinki, Helsinki, Finland; 5 Department of Psychological & Brain Sciences, Indiana University, Bloomington, IN USA; 6 Department of Public Health, University of Turku, Turku, Finland; 7 School of Health Sciences, University of Skövde, Skövde, Sweden

**Keywords:** Sense of coherence, Mental health, Personality, Twins, Polygenic score

## Abstract

**Purpose:**

Sense of coherence (SOC) represents coping and can be considered an essential component of mental health. SOC correlates with mental health and personality, but the background of these associations is poorly understood. We analyzed the role of genetic factors behind the associations of SOC with mental health, self-esteem and personality using genetic twin modeling and polygenic scores (PGS).

**Methods:**

Information on SOC (13-item Orientation of Life Questionnaire), four mental health indicators, self-esteem and personality (NEO Five Factor Inventory Questionnaire) was collected from 1295 Finnish twins at 20–27 years of age.

**Results:**

In men and women, SOC correlated negatively with depression, alexithymia, schizotypal personality and overall mental health problems and positively with self-esteem. For personality factors, neuroticism was associated with weaker SOC and extraversion, agreeableness and conscientiousness with stronger SOC. All these psychological traits were influenced by genetic factors with heritability estimates ranging from 19 to 66%. Genetic and environmental factors explained these associations, but the genetic correlations were generally stronger. The PGS of major depressive disorder was associated with weaker, and the PGS of general risk tolerance with stronger SOC in men, whereas in women the PGS of subjective well-being was associated with stronger SOC and the PGSs of depression and neuroticism with weaker SOC.

**Conclusion:**

Our results indicate that a substantial proportion of genetic variation in SOC is shared with mental health, self-esteem and personality indicators. This suggests that the correlations between these traits reflect a common neurobiological background rather than merely the influence of external stressors.

**Supplementary Information:**

The online version contains supplementary material available at 10.1007/s00127-021-02098-6.

## Introduction

While mental health and well-being are related and even used interchangeably, mental health is generally taken to refer to the presence of psychiatric illness or psychopathology, whereas well-being is about positive psychological health. Thus, a state of health may be regarded not only as the absence of disease but can also cover positive qualities such as life satisfaction, motivation and a sense of fulfilment. Well-being is often characterized by two components: (i) “feeling well” (i.e., hedonic well-being, covering overall interest in life, happiness and life satisfaction) and (ii) “doing well” (i.e., eudaemonic well-being, which represents a more cognitive appraisal of one’s relationships, personal growth and optimal functioning) [1]. The salutogenic approach to well-being relates to “doing well” by emphasizing resources helping to cope with external stressors and factors supporting mental health [2]. A key concept in the salutogenic theory is the sense of coherence (SOC): a life orientation enabling coping in arduous life situations and thus staying healthy regardless of the presence of external stressors. A strong SOC allows for seeing the world as comprehensible, meaningful and manageable, reflecting the three main dimensions of SOC. Previous studies have shown that SOC is associated with better mental and physical health [3] and a better quality of life as predicted by the theory [4]. Further, high internal validity of SOC has been shown in previous studies, as well as moderate to high test–retest correlations, at least within the time frame of a few years, showing the consistency of SOC [5].

Although it has been proposed that SOC represents a fundamentally different approach to mental health measuring the capacity to maintain health (salutogenesis) in contrast to the classical focus on risks and diseases (pathogenesis) [6], previous research has challenged this distinction. There is evidence that SOC is inversely associated with indicators of mental health problems, such as depression and anxiety [7, 8]. It is possible that SOC measures the same dimension of mental health measured by instruments focused on identifying psychiatric diseases and mental health problems. Further, associations have been found between SOC and personality factors: according to a recent meta-analysis of 19 studies using the NEO Five Factor Inventory (FFI) personality scale, neuroticism was associated with weaker and extraversion, openness, agreeableness and conscientiousness with stronger SOC [9].

A limitation in the previous studies on the associations of SOC with mental health and personality indicators is that they have not considered whether the same environmental factors can generally be behind these associations. For example, if the same external exposure, known to be negatively associated with SOC, such as negative life events [10] or work-related stress [11], also affects mental health, it can create a correlation between these traits. These effects can be reinforced since many studies of SOC have been conducted in patients or other populations with special external stressors [3, 4]. Similarly, shared genetic effects may underlie the associations of SOC with other traits. It has been shown that many psychiatric disorders show substantial genetic correlations based on measured genetic variants [12, 13], suggesting that a shared genetic liability may exist. Measures of mental well-being also show substantial heritability in twin and family studies, and some large molecular genetics studies of well-being have been conducted [14, 15]. Nonetheless, progress in identifying the actual genetic basis of well-being is much slower than for specific mental disorders. Thus, it is possible that some of the same genes that explain individual differences in SOC are related to individual differences in mental health problems. Molecular genetic studies of SOC are, however, still lacking.

In this study, we aim to obtain more information on these associations by studying the genetic correlations between SOC, mental health, self-esteem and personality. We will use two approaches to estimate the common genetic background, each making different theoretical assumptions: (i) a classical twin design utilizing the genetic similarity of twins and (ii) a molecular genetic design utilizing measured information on genetic polymorphisms through the whole genome.

## Data and methods

We used data derived from the FinnTwin12 study having the target population of all Finnish twins born in 1983–1987 (*N* = 6272) [16]. From the Finnish population registry (which covers the entire population), the twins were identified as those born on the same day to the same mother. The baseline postal questionnaire was sent to the twins when they were 11 to 12 years of age, and 4920 twins responded (78% of all twins in the cohort). This current study is based on the fourth wave of the FinnTwin12 study, when the twins were 20–27 years of age. During this time, a sub-cohort of twins were invited to an intensive study when information on SOC, mental health, self-esteem and personality was collected by a questionnaire, and they also gave a DNA sample [17]. Together, 1852 twins were invited to this intensive study and 1295 twins returned the questionnaire (54% females). After removing two twin individuals with unknown zygosity, we had 254 complete monozygotic (MZ), 176 same-sex dizygotic (SSDZ), and 156 opposite-sex dizygotic (OSDZ) pairs informing the genetic twin modeling. Twins without information on their co-twin (*N* = 121) were removed from the genetic twin analyses. Further, we had genotypic data available for 1257 twin individuals used in the molecular genetic analyses. For twins with a DNA sample, zygosity was based on measured genotypes (i.e., single-nucleotide polymorphisms (SNP)), and for the few twins who did not give a DNA sample, zygosity was based on questions of physical similarity in the baseline questionnaire, a method that has shown high reliability in this cohort [18]. The number of missing observations for individual items was small (*N* = 41 for schizotypal personality and a maximum of five for other traits).

SOC was measured with the Antonovsky’s 13-item short scale derived from the original 29-item Orientation to Life Questionnaire [2]. The mean score of all items was used as a measure of SOC. The Cronbach’s α for SOC in our data was 0.85, showing good internal consistency of the scale. We decided to only report the results for general SOC because it correlated strongly with all three dimensions of SOC (*r* = 0.89 for comprehensibility, *r* = 0.86 for manageability and *r* = 0.80 for meaningfulness after adjusting for sex) in this sample. However, to confirm that the different dimensions of SOC were not differently associated with other psychological traits, we calculated the trait correlations of them with mental health indicators, self-esteem and personality traits. The heritability estimates for SOC and its three components have previously been reported in a larger cohort that included also twin pairs used in this study [19].

Mental health was measured with four scales described in detail elsewhere. Cronbach’s α values calculated for mental health and self-esteem varied from 0.80 to 0.91, showing good to excellent internal consistency of the scales. Depressive symptoms were measured by the 10-item short version of the General Behavior Inventory questionnaire on mood-related behaviors such as depressive, hypomanic and biphasic symptoms, which was also used in previous Finnish studies (*α* = 0.91) [20]. Alexithymia was measured by the 20-item Toronto Alexithymia Scale measuring problems of describing and identifying emotions [21]. A sum score with a possible range of 20–100 was used (*α* = 0.83). Schizotypal personality was measured by the 22-item Schizotypal Personality Questionnaire—Brief (*α* = 0.80) [22]. Overall mental health problems were measured with the Goldberg 12-item General Health Questionnaire (GHQ) asking about issues affecting general mood and mental health problems in everyday life (*α* = 0.87) [23]. Self-esteem was assessed by the 10-item Rosenberg Self-Esteem Scale, a uni-dimensional measure of global self-esteem measuring overall feelings of self-worth and self-acceptance [24] and previously used in studies based on Finnish twin data [25]. We used the sum score with a possible range of 10–40 (*α* = 0.89). Personality was measured with the 55-item NEO FFI that yielded mean scores in five personality traits: neuroticism, extraversion, openness, agreeableness and conscientiousness [26]. The Finnish version of the NEO FFI is based on a longer 180-item personality inventory, which is an authorized adaptation of the NEO Personality Inventory [27]. Seven extra items for a sensation-seeking facet of the extraversion scale were also used in our study.

We started the analyses by examining genetic and environmental factors affecting SOC, mental health, self-esteem and personality using a quantitative genetic twin design based on the comparisons of similarity between MZ and DZ twins [28]. MZ twins are virtually genetically identical at the gene sequence level whereas DZ twins share half of their genetic variation, similar to ordinary siblings. Both MZ and DZ twins are assumed to share the same amount of environmental variation. Based on these principles, the trait variation can be decomposed to additive genetic variation (A) including all main effects of the loci affecting the trait (correlations of 1.0 within MZ and 0.5 within DZ pairs); dominance genetic variation (D) caused by interactions between alleles in the same locus (correlations of 1.0 within MZ and 0.25 within DZ pairs); shared environment (C) including the effect of all environmental factors that make twins in a pair similar to each other (correlations of 1.0 within both MZ and DZ twins); and unique environment (E) including environmental factors specific to each twin individual as well as any measurement error (correlation of 0 within both MZ and DZ twins). Because we have only twins reared together in our data, we were unable to estimate D and C effects simultaneously.

Univariate models were used to test the assumptions of twin modeling (i.e., the same means and standard deviations (SD) for first- and second-born twins as well as MZ and DZ twins), finding the best fitting model, as well as to estimate the heritability components (i.e., the proportion of total variation explained by genetic variation) under the best fitting model. Since age was not correlated with SOC, the mental health indicators, self-esteem or the personality factors (*r* = − 0.06–0.05; *p* values ≥ 0.064), we did not adjust the results for age. Model fit statistics are presented in Supplementary Table 1. We first estimated the additive genetic/shared environment/unique environment (ACE) model and the additive genetic/dominance genetic/unique environment (ADE) model. When we compared a more parsimonious additive genetic/unique environment (AE) model to the better fitting model (ADE or ACE model having a lower − 2 log likelihood value), we found that the decrease of model fit was not statistically significant (*p* ≥ 0.344), suggesting that shared environment and dominance genetic factors were not needed in the model and could be constrained to zero. Thus, the model used in our study makes assumptions that all effects of alleles on the psychological traits are additive and there are no environmental factors shared by co-twins affecting these traits. Sex-specific genetic factors were statistically significant for four traits and the size of variance components showed statistically significant differences between men and women for six traits. When the full AE model was compared with the saturated model, a violation of the assumptions of twin modeling was found for depression (*p* = 0.050), overall mental health problems (*p* < 0.001), and self-esteem (*p* = 0.035). However, if using the Bonferroni-corrected p values for multiple testing (*p* = 0.0045 for 11 tests based on the conventional significance level of 0.05), the violation was statistically significant only for overall mental health problems. Thus, we used the full AE model to (i) estimate the proportion of variation of SOC, mental health indicators, self-esteem and personality components explained by additive genetic and unique environmental factors using a univariate model and then (ii) calculated how much these factors explained the associations of SOC with the other psychological measures using Cholesky decomposition. This method decomposes all variation and co-variation into uncorrelated latent factors.

We continued the study by analyzing how SOC correlated with the polygenic risk scores (PGS) of the following six traits: major depressive disorder, general risk tolerance, schizophrenia, depression, neuroticism and subjective well-being. The technical details of genotyping have been described elsewhere [29]. To obtain PGSs, we implemented a Bayesian approach taking into account the linkage disequilibrium between each variant [30]. The model for calculating PGSs was adjusted for a linkage disequilibrium reference panel consisting of 27,284 unrelated Finnish samples from the national FINRISK study [31]. Genome-wide-association (GWA) summary statistics for the PGS calculations were obtained from the Psychiatric Genomics Consortium website (https://www.med.unc.edu/pgc/) for major depression [32] and schizophrenia [33], from the Social Science Genetic Association Consortium website (https://www.thessgac.org/data) for general risk tolerance [34] and subjective well-being [14] and from the Neale lab repository of UK Biobank summary statistics for depression and neuroticism scores (http://www.nealelab.is/uk-biobank). The total number of SNPs used for PGS calculations were 1,147,810 for major depression, 1,144,587 for schizophrenia, 1,147,378 for general risk tolerance, 99,7410 for subjective well-being, 1,142,239 for broad depression and 1,142,239 for neuroticism scores. We calculated standardized β-coefficients presenting the change of SOC score per the change of 1 SD of each PGS as well as the proportion of SOC variance explained by each PGS (*R*^2^).

Finally, we conducted a GWA analysis for SOC using the following procedure: genotypes were imputed to the TOPMed release 2 reference panel [35] using minimac4 [36] in the TOPMed imputation server [37] and then we performed association analysis using linear mixed models using age, sex and eight genetic principal components as covariates with an empirical kinship matrix in the random effect of the model controlling for familial and more distant genetic relatedness. The association testing was performed using score tests using genotype dosages for alternate alleles. The analysis was performed using the RVTESTS package [37]. After the analysis, we filtered out all variants with alternate an allele frequency < 1%, HWE *p* value < 1e−06 and imputation quality < 0.8.

The genetic twin models were fitted using the OpenMx package, version 3.0.2, of R statistical software [38]. The PGS analyses as well as the statistical tests for all descriptive statistics were performed using linear regression models by the Stata/SE 16.1 for Windows statistical software (StataCorp, College Station, TX, USA) using the cluster option to correct the standard errors and confidence intervals (CI) for the lack of statistical independence of twins sampled as twin pairs [39].

## Results

Table 1 presents the descriptive statistics for SOC, mental health indicators, self-esteem and personality traits by sex. Men had stronger SOC and generally better mental health and self-esteem than women. The only exception was alexithymia, with men reporting more problems in identifying emotions. In the personality traits, men showed a higher level of extraversion whereas women had higher levels of neuroticism, openness and agreeableness. In conscientiousness, no sex difference was found.

**Table 1:** Descriptive statistics of sense of coherence, mental health, self-esteem and personality factors by sex

	Men			Women			*p*-value of sex difference
	*N*	Mean	SD	*N*	Mean	SD	
Sense of coherence	594	64.1	10.00	700	61.7	10.63	< 0.0001
Mental health							
Depression	593	13.6	4.10	699	15.3	4.94	< 0.0001
Alexithymia	594	29.7	9.42	700	27.8	10.29	0.002
Schizotypal personality	576	5.2	4.32	677	6.1	4.42	0.001
Overall mental health problems	591	21.3	4.24	699	23.5	5.52	< 0.0001
Self-esteem	593	33.2	4.94	699	30.4	5.54	< 0.0001
Personality							
Neuroticism	595	1.4	0.63	700	1.9	0.70	< 0.0001
Extraversion	595	2.5	0.46	700	2.3	0.41	< 0.0001
Openness	595	2.0	0.54	700	2.2	0.51	< 0.0001
Agreeableness	595	2.6	0.42	700	2.7	0.47	0.010
Conscientiousness	595	2.5	0.53	700	2.6	0.54	0.218

Table 2 presents the proportions of total variance decomposed to additive genetic and unique environmental variances for all psychological traits. The results showed moderate heritability estimates varying between 0.19 and 0.66. For most of the traits, men showed lower heritabilities than women. Even when the 95% CIs were overlapping, the model fit statistics showed that the magnitude of genetic effects differed between men and women in depression, overall mental health problems, self-esteem, neuroticism, extraversion and agreeableness (Supplementary Table 1).

**Table 2:** The proportion of variation of sense of coherence, mental health, self-esteem and personality indicators explained by additive genetic and unique environmental factors by sex

	Additive genetic factors			Unique environmental factors		
	a^2^	95% CI		e^2^	95% CI	
		LL	UL		LL	UL
Men						
Sense of coherence	0.30	0.15	0.44	0.70	0.56	0.85
Mental health						
Depression	0.37	0.21	0.51	0.63	0.49	0.79
Alexithymia	0.44	0.29	0.56	0.56	0.44	0.71
Schizotypal personality	0.52	0.38	0.63	0.48	0.37	0.62
Overall mental health problems	0.19	0.02	0.35	0.81	0.65	0.98
Self-esteem	0.35	0.18	0.49	0.65	0.51	0.82
Personality						
Neuroticism	0.43	0.29	0.55	0.57	0.45	0.71
Extraversion	0.60	0.48	0.69	0.40	0.31	0.52
Openness	0.66	0.55	0.74	0.34	0.26	0.45
Agreeableness	0.24	0.09	0.38	0.76	0.62	0.91
Conscientiousness	0.51	0.37	0.62	0.49	0.38	0.63
Women						
Sense of coherence	0.51	0.39	0.61	0.49	0.39	0.61
Mental health						
Depression	0.63	0.52	0.71	0.37	0.29	0.48
Alexithymia	0.41	0.29	0.52	0.59	0.48	0.71
Schizotypal personality	0.57	0.47	0.66	0.43	0.34	0.53
Overall mental health problems	0.40	0.26	0.51	0.60	0.49	0.74
Self-esteem	0.59	0.49	0.68	0.41	0.32	0.51
Personality						
Neuroticism	0.58	0.47	0.66	0.42	0.34	0.53
Extraversion	0.56	0.46	0.65	0.44	0.35	0.54
Openness	0.61	0.51	0.69	0.39	0.31	0.49
Agreeableness	0.57	0.46	0.66	0.43	0.34	0.54
Conscientiousness	0.54	0.42	0.64	0.46	0.36	0.58

The trait correlations of SOC with the other psychological traits as well as the decomposition of these trait correlations to additive genetic and unique environmental correlations are presented in Table 3. Those with stronger SOC had lower levels of depression, alexithymia, schizotypal personality and overall mental health problems and better self-esteem. Generally, the correlations were very similar in men and women. When these trait correlations were decomposed, both additive genetic and unique environmental factors explained a part of them. However, additive genetic correlations were, with a few exceptions, substantially stronger than unique environmental correlations. The strongest genetic correlations were found for depression (*r*_A_ = − 0.82 in men and − 0.84 women), showing that 67% of genetic variation in men and 71% in women was shared with SOC; in females the genetic correlation for overall mental health problems was also high (*r*_A_ = − 0.84). However, all genetic correlations were at least moderate, showing that the traits share 17% or more common genetic variation with SOC.

**Table 3:** Trait correlations of sense of coherence with mental health, self-esteem and personality factors as well as additive genetic and unique environmental correlations explaining these trait correlations by sex

	Trait correlation			Additive genetic correlation				Unique environmental correlation			
	*r*	95% CI		*r*_A_	95% CI		% explained	*r*_E_	95% CI		% explained
		LL	UL		LL	UL			LL	UL	
Men											
Mental health											
Depression	− 0.58	− 0.63	− 0.52	− 0.82	− 1.00	− 0.60	0.48	− 0.46	− 0.57	− 0.32	0.52
Alexithymia	− 0.50	− 0.57	− 0.43	− 0.71	− 0.98	− 0.46	0.51	− 0.39	− 0.52	− 0.25	0.49
Schizotypal personality	− 0.61	− 0.66	− 0.55	− 0.65	− 0.83	− 0.45	0.43	− 0.61	− 0.70	− 0.49	0.57
Overall mental health problems	− 0.47	− 0.53	− 0.40	− 0.41	− 0.85	0.34	0.18	− 0.49	− 0.59	− 0.36	0.82
Self-esteem	0.62	0.56	0.67	0.53	0.13	0.73	0.28	0.66	0.56	0.74	0.72
Personality											
Neuroticism	− 0.69	− 0.73	− 0.64	− 0.78	− 0.93	− 0.59	0.41	− 0.65	− 0.73	− 0.54	0.59
Extraversion	0.32	0.24	0.39	0.39	0.13	0.63	0.52	0.29	0.13	0.44	0.48
Openness	− 0.07	− 0.16	0.02	− 0.15	− 0.41	0.11	0.99	0.00	− 0.17	0.17	0.01
Agreeableness	0.41	0.33	0.48	0.59	0.19	0.96	0.34	0.36	0.22	0.48	0.66
Conscientiousness	0.35	0.27	0.43	0.60	0.34	0.87	0.66	0.21	0.05	0.36	0.34
Women											
Mental health											
Depression	− 0.66	− 0.71	− 0.61	− 0.84	− 0.93	− 0.75	0.72	− 0.43	− 0.55	− 0.30	0.28
Alexithymia	− 0.53	− 0.59	− 0.47	− 0.66	− 0.79	− 0.50	0.56	− 0.43	− 0.54	− 0.30	0.43
Schizotypal personality	− 0.62	− 0.67	− 0.57	− 0.79	− 0.89	− 0.68	0.69	− 0.42	− 0.54	− 0.30	0.31
Overall mental health problems	− 0.55	− 0.60	− 0.49	− 0.84	− 0.98	− 0.69	0.69	− 0.31	− 0.44	− 0.18	0.31
Self-esteem	0.62	0.57	0.67	0.72	0.61	0.83	0.66	0.51	0.39	0.61	0.34
Personality											
Neuroticism	− 0.72	− 0.76	− 0.68	− 0.85	− 0.93	− 0.76	0.63	− 0.59	− 0.68	− 0.48	0.37
Extraversion	0.17	0.09	0.25	0.12	− 0.07	0.30	0.37	0.23	0.08	0.37	0.63
Openness	− 0.07	− 0.15	0.01	− 0.17	− 0.35	0.01	1.31	0.05	− 0.10	0.20	− 0.31
Agreeableness	0.42	0.35	0.49	0.70	0.55	0.85	0.88	0.11	− 0.04	0.25	0.12
Conscientiousness	0.33	0.25	0.40	0.52	0.34	0.70	0.81	0.13	− 0.03	0.28	0.19

When studying the personality traits, those men and women with stronger SOC showed a lower level of neuroticism but higher levels of extraversion, agreeableness and conscientiousness. For openness, no association with SOC was found. As for the mental health traits and self-esteem, genetic correlations were higher than unique environmental correlations even when both of these components explained a share of co-variation between SOC and personality. The highest genetic correlation of SOC was found with neuroticism (*r*_A_ = − 0.78 in men and *r*_A_ = − 0.85 in women) showing that 61% of genetic variation in men and 72% in women was shared between these traits. When we tested the associations of mental health traits, self-esteem and personality factors separately with the three dimensions of SOC, we found that the correlations were very similar for comprehensibility, manageability and meaningfulness (Supplementary Table 2).

Table 4 presents the associations of the PGSs of three mental health disorders, general risk tolerance, subjective well-being and neuroticism with SOC. In men, the PGS of major depressive disorders was associated with weaker and general risk tolerance with stronger SOC; for schizophrenia a negative association was found, but it was only marginally significant. In women, the PGSs of depression and neuroticism were associated with weaker and subjective well-being with stronger SOC. We also tested the sex interactions for all PGSs, but they were not significant (*p* ≥ 0.278). The PGSs explained from 2 to 6% of the SOC variation.

**Table 4:** The standardized regression coefficients and the proportions of variation explained by polygenic scores for sense of coherence

	Men				Women			
	β	95% CI		*R*^2^	β	95% CI		*R*^2^
		LL	UL			LL	UL	
Major depressive disorder	− 1.06	− 1.97	− 0.16	0.06	− 0.72	− 1.53	0.09	0.03
General risk tolerance	1.03	0.19	1.86	0.06	− 0.39	− 1.24	0.45	0.03
Schizophrenia	− 0.81	− 1.62	0.01	0.05	− 0.09	− 1.00	0.81	0.02
Depression	0.17	− 0.68	1.01	0.05	− 0.82	− 1.59	− 0.06	0.03
Neuroticism	− 0.26	− 1.14	0.61	0.05	− 0.86	− 1.67	− 0.05	0.03
Subjective well-being	− 0.15	− 1.12	0.81	0.05	0.93	0.10	1.75	0.03

**Fig. 1:**
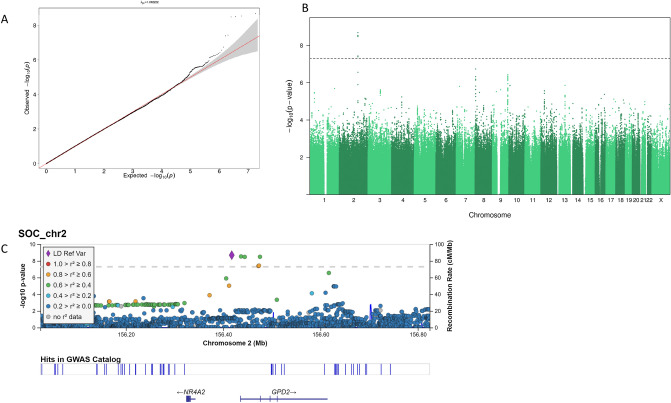
The genome-wide scan of sense of coherence. **a** Quantile–quantile plot of all *p* values from variants passing quality control with a 45° reference line plotted. A genomic inflation factor of λ = 1.00 shows no inflation in *p* values caused by population stratification or sample relatedness. **b** Manhattan plot of *p* values from variants passing quality control. Horizontal line indicates genome-wide significant threshold for *p* values (5e−08). **c** Regional plot of chromosome 2 results showing linkage disequilibrium between lead-SNP (rs74920024) and other variants within the zoomed region. The plot was generated with LocusZoom (https://my.locuszoom.org/)

Finally, we conducted the genome-wide scan of SOC on 1257 twin individuals. This analysis revealed four genome-wide significant associations, all nearby on chromosome 2, for the following SNPs (rs74920024, rs72893369, rs148317278, rs72893377) with *p* values from 2.034e−09 to 3.300e−09. The rs74920024 reference allele is G, while the minor allele frequency of the A-allele is 0.02277. The Q–Q plot is shown in Fig. 1a and the Manhattan plot in Fig. 1b. The locus zoom plot (Fig. 1c) shows the locally correlated SNPs, but after conditioning on the top SNP, no further genome-wide signals were seen. The closest gene is glycerol-3-phosphate dehydrogenase 2 (GPD2). The GWA catalog reports no associations for the top SNP (https://www.ebi.ac.uk/gwas/home), but there are hits close by (Fig. 1c).

## Discussion

Our results based on a Finnish sample of twins demonstrated that genetic factors explained a moderate proportion of individual differences in SOC, mental health, self-esteem and personality. These estimates corresponded well with the previous studies. Generally similar heritability estimates of personality [40] as well as mental health indicators used in this study, such as alexithymia [41] and depressive symptoms [42], have been previously reported. The previously reported heritability of SOC in a larger cohort of twins from which this cohort was derived was also very similar [19]. These results thus support the robustness of the genetic architecture of these psychological traits in adulthood with moderate heritability.

We found that SOC was associated with mental health indicators, self-esteem and personality traits. SOC was negatively associated with depression, alexithymia, schizotypal personality and overall mental health problems and positively associated with self-esteem. Previous studies have reported positive associations of SOC with life satisfaction [8] and negative associations with depression and anxiety [7, 8]. Additionally, for personality, the results were similar to those of previous studies. A recent meta-analysis of 24 independent samples (19,960 participants) using the NEO FFI personality scale found that, in accordance with our study, neuroticism was associated with weaker and extraversion, agreeableness and conscientiousness with stronger SOC. This meta-analysis also reported that openness was associated with stronger SOC, a finding we could not replicate; however, this correlation was substantially lower than correlations with other personality traits [9].

Our most novel results concerned the role of genetic factors behind the associations of SOC with the other psychological traits. Our results based on the genetic twin design revealed that even when both genetic and unique environmental factors contributed to these associations, the genetic correlations were generally stronger than unique environmental correlations. There was substantial variation in the size of genetic correlations, but they showed that from 30 to 60% of the genetic variation of most mental health indicators, self-esteem and personality traits was shared with SOC. On the other hand, this also shows that since the proportions are much less than 100%, there are also genetic factors affecting these traits independently. The role of genetic factors was confirmed by the molecular genetic results showing that the PGSs of mental health indicators were correlated with SOC. The variation of SOC explained by the PGSs was 6% or less. However, this much lower share of genetic variation than found when using genetic twin modeling is expected since the PGSs do not yet cover the whole genome level variation and subsequently explain only a fraction of the genetic variation of mental health indictors estimated by genetic twin modeling, a phenomenon known as missing heritability [43]. For example, the heritability of neuroticism based on the PGS (called as SNP heritability) is only 0.11 (http://www.nealelab.is/uk-biobank), while the heritability estimates of neuroticism were 0.43 in men and 0.58 in women in our data estimated using the genetic twin modeling. Considering the difference between SNP heritability and heritability estimated based on genetic twin modeling, our results on shared genetic variation based on PGSs and genetic twin modeling are consistent. Since these two methods to estimate genetic variance are based on totally different theoretical assumptions, together they provide strong evidence for shared genetic effects between SOC and a range of mental health and personality traits.

This evidence of overlap in genetic liability to individual variation in SOC with psychological well-being, dimensions of mental health and personality creates opportunities for further research. The search for endophenotypes that mediate effects of dispositional genes on observed phenotypes of psychopathology [44] can be extended to endophenotypic pathways for risk-relevant behavioral precursors as illustrated by a study suggesting a novel metabolic biomarker, ketone body 3-hydroxybutyrate, of aggression [45]. Such research is responsive to the call to create a holistic framework for enhanced understanding of mental health traits, proposed by the National Institute of Mental Health’s Research Domain Criteria (RDoC) Initiative [46]. Another part of the holistic framework creation goal of the RDoC initiative would be the elaboration of models of well-being [47]. Our results suggest the possibility of identifying biobehavioral processes that underlie broad dimensions of psychological health, ranging from processes creating high risk for psychiatric disorders to less severe, but more common behavioral and adjustment problems, to individual differences in resilience to stress, self-efficacy, and positive psychological health. Identifying possible endophenotypes underlying diverse mental health traits and effectively fostering dimensional research on the biobehavioral matrix of mental health and mental illness is a challenging new opportunity for multidisciplinary research.

Our GWA analysis, to our knowledge the first one conducted for SOC, yielded a significant association (*p* = 2.034e−09 for rs74920024 on chromosome 2), with three other significant associations, though just one independent signal. While our study is a priori underpowered with a sample size of 1257, the use of a continuous variable and the more powerful TOPMed reference panel may have partly compensated for this problem. The association locus is near but not in the closest gene, GPD2. The gene encodes a protein that localizes to the inner mitochondrial membrane and is responsible for the conversion of glycerol-3-phosphate to dihydroxyacetone phosphate. Interestingly, it has been associated in several large GWA analyses with education [48] and cognitive ability [49] in addition to myopia [50], a condition known to be aggravated by reading. However, because of the small sample size for the GWA, our results should be considered as only preliminary and require replications in other datasets.

There can be different mechanisms explaining the genetic correlations found. First, they can reflect the influence of genes on different traits (called pleiotropy). Second, they can be because of causal associations between the traits. For example, if strong SOC is causally associated with better self-esteem, it can create a genetic correlation when genetic polymorphisms increasing SOC also explain better mental health. However, the fact that genetic correlations were systematically higher than the trait correlations does not support this explanation. Third, the genetic correlations can indicate the same neurobiological background behind these indicators. We found this explanation most plausible, and it is consistent with the theory that SOC largely captures the same dimension of mental health as other known indicators of mental health [51].

Since the prevalence of internalized mental health disorders is higher in women than in men [52], we stratified all analyses by sex to study whether sex differences may also exist in the associations between SOC and other psychological traits. We found that, except alexithymia, men had better mental health and also higher self-esteem than women. Additionally, SOC was stronger in men than in women, as also found in previous studies [53, 54]. Consistent with previous studies [55], men had higher levels of extraversion and women higher levels of neuroticism, openness and agreeableness. Considering these mean differences, it is interesting that the associations of SOC with mental health, self-esteem and personality traits were very similar in men and women, and we did not find any systematic sex differences in the genetic correlations either. When using PGSs, some sex differences were found, but they were not statistically significant and, therefore, can also be because of sample error. Thus, based on this study, the genetic background of SOC and other psychological traits seems to be broadly similar in men and women.

Our study has both strengths and weaknesses. Our main strengths were that we had information on SOC and several mental health, well-being and personality indicators measured with well-validated questionnaires in genetically informative data. We were able to study the common genetic background of SOC, mental health, self-esteem and personality using two different approaches—the classical twin design utilizing information on the similarity of MZ and DZ twins and the molecular genetic design utilizing measured genetic polymorphisms—each making different theoretical assumptions. The robustness of these results provides more convincing evidence on the role of genes behind these associations. A limitation is that we needed to rely on self-reported data, without information on clinically validated psychiatric diseases or ratings of personality factors by others. Thus, a response bias may have strengthened trait correlations, but they are not likely to explain our main results (i.e., the genetic correlations between the traits). Finally, it is clear that our data are underpowered for GWA; thus, these results need to be considered as preliminary, needing replications in other datasets.

In conclusion, we found that SOC shares a substantial proportion of genetic variation with mental health indicators, self-esteem and personality factors. This indicates that the correlations between these traits reflect in part a common neurobiological background rather than the influence of external stressors. Our results may suggest that mental health forms a continuum from mental health disorders of various severity to the positive end of mental health and well-being. Further research is needed to find neurobiological mechanisms behind the whole variation of mental health. Our results also suggest that mental health treatment may benefit a larger share of the population than only those with diagnosed mental health disorders.

## Supplementary Information

Below is the link to the electronic supplementary material.

Supplementary file 1 (DOCX 39 KB)

## Declarations

### Availability of data and materials

Because of the consent given by study participants and the high degree of identifiability, data cannot be made publicly available. Data are available through the Institute for Molecular Medicine Finland (FIMM) Data Access Committee (DAC) for authorized researchers who have IRB/ethics approval and an institutionally approved study plan. For more details, please contact the FIMM DAC (fimm‐dac@ helsinki.fi).

### Code availability

All computer codes are available from the corresponding author.
