# Supplementary material for: The genetic background of the associations between sense of coherence and mental health, self-esteem and personality
Source: Soc Psychiatry Psychiatr Epidemiol. 2021 May 19;57(2):423–33. doi: 10.1007/s00127-021-02098-6 (PMC8602419; doi:10.1007/s00127-021-02098-6)
Supplement: Supplementary file 1 — (DOCX 39 KB) [file 127_2021_2098_MOESM1_ESM.docx]

Supplementary table 1. Model fit statistics of genetic twin modeling for sense of coherence, mental health, self-esteem and personality factors.

|  | **-2 log likelihood** | | | | **Model comparison statistics for AE model** | | | | | | | |
| --- | --- | --- | --- | --- | --- | --- | --- | --- | --- | --- | --- | --- |
|  | **Saturated model** | **Full ACE model** | **Full**  **ADE model** | **Full**  **AE model** | **No sex-specific genetic effect** | | **Same parameters for men and women** | | **Full AE model compared to ACE/ADE model** | | **Full AE model compared to saturated model** | |
|  |  |  |  |  | **Δ**  **-2LL** | **p-value^1^** | **Δ**  **-2LL** | **p-value^2^** | **Δ**  **-2LL** | **p-value^3^** | **Δ**  **-2LL** | **p-value^4^** |
| **Sense of coherence** | 8717.9 | 8737.9 | 8736.9 | 8738.0 | 5.6 | 0.018 | 5.7 | 0.059 | 1.1 | 0.773 | 20.0 | 0.330 |
| **Mental health** |  |  |  |  |  |  |  |  |  |  |  |  |
| Depression | 6756.3 | 6785.1 | 6782.5 | 6785.1 | 0.6 | 0.429 | 22.6 | <0.001 | 2.6 | 0.276 | 28.8 | 0.050 |
| Alexithymia | 8597.9 | 8613.4 | 8613.4 | 8613.4 | 0.9 | 0.340 | 2.0 | 0.369 | 0.0 | 0.999 | 15.5 | 0.626 |
| Schizotypal personality | 6474.9 | 6490.8 | 6492.2 | 6492.2 | 0.8 | 0.371 | 0.4 | 0.802 | 1.4 | 0.499 | 17.3 | 0.500 |
| Overall mental health problems | 6921.3 | 6965.9 | 6965.0 | 6965.9 | 5.3 | 0.021 | 40.3 | <0.001 | 0.0 | 0.991 | 44.6 | <0.001 |
| **Self-esteem** | 7064.0 | 7094.3 | 7092.9 | 7094.3 | 0.3 | 0.562 | 10.9 | 0.004 | 1.3 | 0.722 | 30.3 | 0.035 |
| **Personality** |  |  |  |  |  |  |  |  |  |  |  |  |
| Neuroticism | 2277.4 | 2301.4 | 2300.7 | 2301.4 | 0.8 | 0.376 | 6.2 | 0.044 | 0.7 | 0.866 | 24.0 | 0.154 |
| Extraversion | 1178.6 | 1198.3 | 1196.7 | 1198.3 | 0.0 | 0.872 | 8.5 | 0.015 | 1.7 | 0.642 | 19.7 | 0.350 |
| Openness | 1614.7 | 1629.4 | 1629.8 | 1629.8 | 42.6 | <0.001 | 1.1 | 0.580 | 0.0 | 1.000 | 15.1 | 0.657 |
| Agreeableness | 1333.9 | 1354.3 | 1351.7 | 1354.5 | 0.3 | 0.602 | 16.5 | <0.001 | 2.8 | 0.425 | 20.6 | 0.298 |
| Conscientiousness | 1770.4 | 1789.7 | 1786.8 | 1790.2 | 18.4 | <0.001 | 0.2 | 0.913 | 3.3 | 0.344 | 19.7 | 0.348 |

^1^Compared to full AE model (Δ d.f.=1); ^2^Compared to full AE model (Δ d.f.=2); ^3^Compared to ADE or ACE depending on a model fit (Δ d.f.=2 if comparing to ACE model and Δ d.f.=3 if comparing to ADE model); ^4^Compared to the saturated model (Δ d.f.=18)

Supplementary table 2. Trait correlations of the dimensions of sense of coherence with mental health, self-esteem and personality factors by sex.

|  | **Comprehensibility** | | | **Manageability** | | | **Meaningfulness** | | |
| --- | --- | --- | --- | --- | --- | --- | --- | --- | --- |
|  | **r** | **95% CI** | | **r** | **95% CI** | | **r** | **95% CI** | |
|  |  | **LL** | **UL** |  | **LL** | **UL** |  | **LL** | **UL** |
| **Men** |  |  |  |  |  |  |  |  |  |
| **Mental health** |  |  |  |  |  |  |  |  |  |
| Depression | -0.50 | -0.56 | -0.44 | -0.48 | -0.54 | -0.41 | -0.48 | -0.54 | -0.42 |
| Alexithymia | -0.47 | -0.53 | -0.41 | -0.36 | -0.43 | -0.29 | -0.44 | -0.50 | -0.37 |
| Schizotypal personality | -0.51 | -0.57 | -0.45 | -0.50 | -0.56 | -0.43 | -0.53 | -0.59 | -0.47 |
| Overall mental health problems | -0.40 | -0.47 | -0.33 | -0.35 | -0.42 | -0.28 | -0.42 | -0.48 | -0.35 |
| **Self-esteem** | 0.51 | 0.45 | 0.57 | 0.50 | 0.43 | 0.56 | 0.55 | 0.49 | 0.61 |
| **Personality** |  |  |  |  |  |  |  |  |  |
| Neuroticism | -0.64 | -0.69 | -0.59 | -0.56 | -0.62 | -0.51 | -0.53 | -0.59 | -0.47 |
| Extraversion | 0.23 | 0.16 | 0.31 | 0.21 | 0.13 | 0.29 | 0.36 | 0.29 | 0.43 |
| Openness | -0.08 | -0.16 | 0.00 | -0.09 | -0.17 | -0.01 | -0.01 | -0.09 | 0.07 |
| Agreeableness | 0.28 | 0.20 | 0.35 | 0.29 | 0.21 | 0.36 | 0.39 | 0.32 | 0.45 |
| Conscientiousness | 0.23 | 0.16 | 0.31 | 0.19 | 0.11 | 0.27 | 0.46 | 0.40 | 0.52 |
| **Women** |  |  |  |  |  |  |  |  |  |
| **Mental health** |  |  |  |  |  |  |  |  |  |
| Depression | -0.55 | -0.60 | -0.50 | -0.56 | -0.61 | -0.51 | -0.56 | -0.61 | -0.51 |
| Alexithymia | -0.48 | -0.54 | -0.43 | -0.44 | -0.50 | -0.38 | -0.46 | -0.51 | -0.40 |
| Schizotypal personality | -0.52 | -0.57 | -0.46 | -0.53 | -0.58 | -0.47 | -0.54 | -0.59 | -0.48 |
| Overall mental health problems | -0.45 | -0.51 | -0.39 | -0.44 | -0.50 | -0.38 | -0.50 | -0.55 | -0.44 |
| **Self-esteem** | 0.53 | 0.47 | 0.58 | 0.54 | 0.48 | 0.59 | 0.56 | 0.50 | 0.61 |
| **Personality** |  |  |  |  |  |  |  |  |  |
| Neuroticism | -0.66 | -0.70 | -0.62 | -0.63 | -0.67 | -0.58 | -0.57 | -0.62 | -0.52 |
| Extraversion | 0.10 | 0.03 | 0.17 | 0.15 | 0.07 | 0.22 | 0.19 | 0.12 | 0.27 |
| Openness | -0.10 | -0.17 | -0.03 | -0.05 | -0.12 | 0.03 | -0.03 | -0.10 | 0.05 |
| Agreeableness | 0.33 | 0.26 | 0.40 | 0.39 | 0.32 | 0.45 | 0.36 | 0.29 | 0.42 |
| Conscientiousness | 0.27 | 0.20 | 0.34 | 0.22 | 0.15 | 0.29 | 0.38 | 0.32 | 0.44 |
