# Supplementary material for: The genetic background of the associations between sense of coherence and mental health, self-esteem and personality
Source: Soc Psychiatry Psychiatr Epidemiol. Author manuscript; Available in PMC 2023 Feb 1. (PMC8602419; doi:10.1007/s00127-021-02098-6)
Supplement: Supplementary file 1 [file NIHMS1706940-supplement-Supplementary_Information.pdf]

Supplementary table 1. Model fit statistics of genetic twin modeling for sense of coherence, mental health, self-esteem and personality factors.

	**-2 log likelihood**				**Model comparison statistics for AE model**							
	**Saturated model**	**Full ACE model**	**Full ADE model**	**Full AE model**	**No sex-specific genetic effect**		**Same parameters for men and women**		**Full AE model compared to ACE/ADE model**		**Full AE model compared to saturated model**	
					**Δ -2LL**	**p-value[1]**	**Δ -2LL**	**p-value[2]**	**Δ -2LL**	**p-value[3]**	**Δ -2LL**	**p-value[4]**
**Sense of coherence**	8717.9	8737.9	8736.9	8738.0	5.6	0.018	5.7	0.059	1.1	0.773	20.0	0.330
**Mental health**												
Depression	6756.3	6785.1	6782.5	6785.1	0.6	0.429	22.6	<0.001	2.6	0.276	28.8	0.050
Alexithymia	8597.9	8613.4	8613.4	8613.4	0.9	0.340	2.0	0.369	0.0	0.999	15.5	0.626
Schizotypal personality	6474.9	6490.8	6492.2	6492.2	0.8	0.371	0.4	0.802	1.4	0.499	17.3	0.500
Overall mental health problems	6921.3	6965.9	6965.0	6965.9	5.3	0.021	40.3	<0.001	0.0	0.991	44.6	<0.001
**Self-esteem**	7064.0	7094.3	7092.9	7094.3	0.3	0.562	10.9	0.004	1.3	0.722	30.3	0.035
**Personality**												
Neuroticism	2277.4	2301.4	2300.7	2301.4	0.8	0.376	6.2	0.044	0.7	0.866	24.0	0.154
Extraversion	1178.6	1198.3	1196.7	1198.3	0.0	0.872	8.5	0.015	1.7	0.642	19.7	0.350
Openness	1614.7	1629.4	1629.8	1629.8	42.6	<0.001	1.1	0.580	0.0	1.000	15.1	0.657
Agreeableness	1333.9	1354.3	1351.7	1354.5	0.3	0.602	16.5	<0.001	2.8	0.425	20.6	0.298
Conscientiousness	1770.4	1789.7	1786.8	1790.2	18.4	<0.001	0.2	0.913	3.3	0.344	19.7	0.348

[1]Compared to full AE model (Δ d.f.=1); [2]Compared to full AE model (Δ d.f.=2); [3]Compared to ADE or ACE depending on a model fit (Δ d.f.=2 if comparing to ACE model and Δ d.f.=3 if comparing to ADE model); [4]Compared to the saturated model (Δ d.f.=18)

Supplementary table 2. Trait correlations of the dimensions of sense of coherence with mental health, self-esteem and personality factors by sex.

	Comprehensibility			Manageability			Meaningfulness		
	r	95% CI		r	95% CI		r	95% CI	
		LL	UL		LL	UL		LL	UL
**Men**									
**Mental health**									
Depression	-0.50	-0.56	-0.44	-0.48	-0.54	-0.41	-0.48	-0.54	-0.42
Alexithymia	-0.47	-0.53	-0.41	-0.36	-0.43	-0.29	-0.44	-0.50	-0.37
Schizotypal personality	-0.51	-0.57	-0.45	-0.50	-0.56	-0.43	-0.53	-0.59	-0.47
Overall mental health problems	-0.40	-0.47	-0.33	-0.35	-0.42	-0.28	-0.42	-0.48	-0.35
**Self-esteem**	0.51	0.45	0.57	0.50	0.43	0.56	0.55	0.49	0.61
**Personality**									
Neuroticism	-0.64	-0.69	-0.59	-0.56	-0.62	-0.51	-0.53	-0.59	-0.47
Extraversion	0.23	0.16	0.31	0.21	0.13	0.29	0.36	0.29	0.43
Openness	-0.08	-0.16	0.00	-0.09	-0.17	-0.01	-0.01	-0.09	0.07
Agreeableness	0.28	0.20	0.35	0.29	0.21	0.36	0.39	0.32	0.45
Conscientiousness	0.23	0.16	0.31	0.19	0.11	0.27	0.46	0.40	0.52
**Women**									
**Mental health**									
Depression	-0.55	-0.60	-0.50	-0.56	-0.61	-0.51	-0.56	-0.61	-0.51
Alexithymia	-0.48	-0.54	-0.43	-0.44	-0.50	-0.38	-0.46	-0.51	-0.40
Schizotypal personality	-0.52	-0.57	-0.46	-0.53	-0.58	-0.47	-0.54	-0.59	-0.48
Overall mental health problems	-0.45	-0.51	-0.39	-0.44	-0.50	-0.38	-0.50	-0.55	-0.44
**Self-esteem**	0.53	0.47	0.58	0.54	0.48	0.59	0.56	0.50	0.61
**Personality**									
Neuroticism	-0.66	-0.70	-0.62	-0.63	-0.67	-0.58	-0.57	-0.62	-0.52
Extraversion	0.10	0.03	0.17	0.15	0.07	0.22	0.19	0.12	0.27
Openness	-0.10	-0.17	-0.03	-0.05	-0.12	0.03	-0.03	-0.10	0.05
Agreeableness	0.33	0.26	0.40	0.39	0.32	0.45	0.36	0.29	0.42
Conscientiousness	0.27	0.20	0.34	0.22	0.15	0.29	0.38	0.32	0.44
